# Interplay between Risk Factors and Coronary Artery Calcium in Middle-Aged and Elderly Symptomatic Patients

**DOI:** 10.31083/j.rcm2406158

**Published:** 2023-06-06

**Authors:** Lu Zeng, Jun-Yi Luo, Fen Liu, Zhuo-Ran Zhang, Ya-Jing Qiu, Fan Luo, Xin-Xin Tian, Xiao-Mei Li, Yi-Ning Yang

**Affiliations:** ^1^Department of Cardiology, The First Affiliated Hospital of Xinjiang Medical University, 830054 Urumqi, Xinjiang, China; ^2^State Key Laboratory of Pathogenesis, Prevention and Treatment of High Incidence Diseases in Central Asia, Clinical Medical Research Institute, The First Affiliated Hospital of Xinjiang Medical University, 830011 Urumqi, Xinjiang, China; ^3^Department of Cardiology, People’s Hospital of Xinjiang Uygur Autonomous Region, 830054 Urumqi, Xinjiang, China

**Keywords:** coronary computed tomography angiography, cardiovascular disease risk factors, coronary artery disease, coronary artery calcium, non-obstructive disease, obstructive disease, middle-aged and elderly patients

## Abstract

**Background:**

The prognostic value of coronary artery calcium (CAC) combined with risk factor burdens in middle-aged and elderly patients with symptoms is unclear.

**Methods:**

A cohort study comprising 7432 middle-aged and elderly symptomatic patients (aged above 55 years) was conducted between December 2013 and September 2020. All patients had undergone coronary computed tomography angiography, and the Agatston score were used to measure CAC scores. The primary outcome was major adverse cardiac and cerebrovascular events (MACCE), which was defined as a composite outcome of nonfatal myocardial infarction, revascularization (percutaneous coronary intervention or coronary artery bypass graft), stroke, and cardiovascular death. Congestive heart failure, cardiogenic shock, malignant arrhythmia, and all-cause mortality were defined as the secondary outcomes.

**Results:**

There are 970 (13%) patients with CAC 0–10, 2331 (31%) patients with CAC 11–100, and 4131 (56%) patients with CAC ≥101. The proportion of patients aged 55–65 years, 65–75 years and ≥75 years was 40.7%, 38.1% and 21.2%, respectively. The total number of MACCEs over the 3.4 years follow-up period was 478. The percentage of CAC ≥101 was higher among the 75-year-old group than the 55–65-year-old group, increasing from 46.5% to 68.2%. With the increase in the CAC score, the proportion of patients aged ≥75 years increased from 12.9% to 25.8%, compared to those aged 55–65 years. The number of risk factors gradually increased as the CAC scores increased in the symptomatic patients aged over 55 years and the similar tendencies were observed among the different age subgroups. The proportion of non-obstructive coronary artery disease (CAD) was comparable between the three age groups (53.5% *vs* 51.9% *vs* 49.1%), but obstruction CAD increased with age. The incidence of MACCE in the group with CAC ≥101 and ≥4 risk factors was 1.71 times higher (95% confidence interval (CI) 1.01–2.92; *p* = 0.044) than the rate in the group with CAC ≥101 and 1 risk factor. In the CAC 0–10 group, the incidence of MACCE in patients aged ≥75 years was 12.65 times higher (95% CI: 6.74–23.75; *p *< 0.0001) than that in patients aged 55–65 years. By taking into account the combination of CAC score, age, and risk factor burden, the predictive power of MACCE can be increased (area under the curve (AUC) = 0.614).

**Conclusions:**

In symptomatic patients aged 55 or above, a rise in age, CAC scores, and risk factor burden was linked to a considerable risk of future MACCE. In addition, combining CAC scores, age and risk factors can more accurately predict outcomes for middle-aged and elderly patients with symptoms.

## 1. Introduction

Cardiovascular disease (CVD) is a major contributor to the worldwide disease burden with a high mortality and disability rate [1]. Cardiovascular diseases include coronary artery disease (CAD), stroke, and peripheral arterial disease [2], wherein CAD is the major contributor to CVD [3]. As the world population is rapidly aging, CVD is becoming more prominent. The 2021 Chinese population census revealed that 17.8% of the population was composed of middle-aged and elderly individuals. As CAD majorly affects the older generation, earlier detection of CAD in the middle-aged and elderly population is crucial [4]. Coronary artery disease is positively and independently linked with the various traditional risk factors, i.e., smoking, diabetes, and obesity [5, 6, 7]. At present, the Chinese population’s exposure to risk factors is universal. For example, the prevalence of hypertension, diabetes, and dyslipidemia was as high as 46.4% in 2018, 10.9% in 2013, and 40.4% in 2012 respectively, compared to 27.9% in 2015, 0.67% in the 1980s, and 18.6% in 2002 [8, 9]. In 2015, 52.1% of Chinese men and 2.7% of women smoked and the total number of smokers was 316 million [10]. It is unfortunate that the existing risk factors are inadequate to provide an accurate prognosis for CVD patients, necessitating further indicators [11].

Coronary computed tomography angiography (CTA) has become a popular imaging technique to diagnose CAD [12, 13]. Moreover, it serves as an important risk stratification tool, especially for symptomatic patients diagnosed with CAD. Coronary CTA can not only identify the coronary plaque features linked to Acute Coronary Syndromes (ACS), but it can also calculate the coronary plaque score. Coronary plaques with ACS had a higher fibro-fatty content and a larger necrotic core volume. Cardiovascular risk rises when fibro-fatty content and necrotic core volume rise [14]. Coronary plaque score included (1) segmental stenosis score; (2) segmental involvement score; (3) plaque score of 3 vessels [15]. Its various advantages include its ability to obtain high-precision results and non-invasive methodology. When performing coronary CTA, the coronary artery calcium (CAC) score, as well as the presence and absence of coronary stenosis, are commonly obtained. Several studies have suggested that CAC can be utilized to evaluate the risk and outcomes of CAD [16, 17, 18]. A high CAC score is linked to a higher risk of cardiovascular events, whereas a low CAC score indicates a lower risk of cardiovascular events. Therefore, coronary CTA has been utilized to determine and classify an individual’s probability of developing CAD. Noticeably, when CAC is united with hematological indices or radiographic indicators, such as high-sensitivity C-reactive protein, low density lipoprotein cholesterol (LDL-C), and pericardial adipose tissue could increase its capacity to predict cardiovascular events [19, 20, 21]. It has been reported that certain risk factors, including body mass index, LDL-C, and smoking, are associated with increased CAC levels [22] and subsequently, a higher occurrence of cardiovascular events [23]. Nevertheless, there have been only a handful of studies that have investigated the relevance of CAC in combination with risk factors. The results of the preceding investigation suggest that CAC combined with other risk factors could improve the assessment of CAD risk in young patients [24]. To our knowledge, there has been no previous studies have focused on utilizing CAC in combination with risk factors to predict prognosis of symptomatic middle-aged and elderly patients.

In this study, we investigated the association between CAC and CAD risk factors and explored the prognostic value of CAC combined with risk factors in symptomatic middle-aged and elderly patients.

## 2. Methods

### 2.1 Patients

Data were obtained from the First Affiliated Hospital of Xinjiang Medical University. Between December 27, 2013, and September 30, 2020, a total of 12,904 symptomatic patients showing signs of CAD were continuously enrolled. The main symptoms of CAD are typical angina or atypical symptoms such as exertional dyspnea or episodes of chest pain at rest, which patients with CAD often experienced. All patients had undergone CTA with symptoms of suspected CAD as a first-line diagnostic imaging and had not received percutaneous coronary intervention (PCI) or coronary artery bypass graft (CABG). Patients with a history of PCI, CABG surgery, tumor, skin disease, immune disease, stroke, pulmonary embolism, infection status, and incomplete outcome data were excluded. The study was approved by the First Affiliated Hospital of Xinjiang Medical University institutional review board (K202106-02), and written informed consent was obtained from all the enrolled patients.

### 2.2 CTA Imaging

Those CTA were uniformly acquired by using multi-detector row computed tomography (CT) scanners consisting of 64-rows or greater (Somatom Definition or Somatom Definition Flash, Siemens Healthcare, Forchheim, Germany). CAC scores was calculated using Agatston’s scoring system. CAC scores were categorized into the following groups: 0–10, 11–100, and ≥101 [25, 26]. Coronary plaques were defined as lesions >1 ${\mathrm{mm}}^{2}$ within and/or adjacent to the coronary artery lumen, which were clearly distinguished from the vessel walls and the pericardium [27]. Plaques were classified according to phenotype: (1) noncalcified plaques (plaques having a lower density compared with the contrast-enhanced vessel lumen); (2) calcified plaques (high-density plaques); and (3) mixed plaques (noncalcified and calcified components within a single plaque). Plaques that exist in both calcified and noncalcified segments were classified as calcified plaques [27].

Prior to the vascular test, the patients had to stay in a reclined position for at least five minutes to maintain stable heart rate values. During coronary CTA acquisition, iodinated contrast (0.8 mL/kg) was delivered intravenously at a steady rate of 4–8 mL/s, with an intravascular contrast agent residence period of no less than 12 s followed by a 30–40 mL saline flush. Stenosis severity was categorized using the quantitative stenosis grading recommended by the Society of Cardiovascular Computed Tomography guidelines [28], it was classified as none (0% luminal stenosis), non-obstructive (1–49% luminal stenosis), or obstructive (≥50% luminal stenosis).

### 2.3 CAD Severity

The degree of coronary stenosis was classified as normal (no coronary stenosis), nonobstructive CAD (lesions <50%), and obstructive CAD (lesions ≥50%). Obstructive CAD was further subdivided into 1-, 2-, and 3-vessel obstructive CAD.

### 2.4 CAD Risk Factors

The following risk factors for coronary artery disease were considered: (1) current smoking status; (2) lipid status, i.e., total cholesterol levels >5.2 mmol/L prior to CTA; (3) triglyceride >1.69 mmol/L prior to CTA; (4) LDL >3.8 mmol/L prior to CTA; (5) high-density lipoprotein (HDL) <1.55 mmol/L prior to CTA; (6) systolic pressure ≥140 mmHg; (7) diastolic pressure ≥90 mmHg prior to CTA; (8) body mass index; (9) diabetes mellitus; (10) family history of CAD (defined as the presence of a first-degree relative with a CAD).

### 2.5 Patient Follow-Up and Outcome

Clinical data were obtained through telephonic follow-ups. A total of 803 patients were lost at follow-up, after reviewing the medical records, these patients had no record of re-visits in our hospital. Participants were followed up every 6–12 months for endpoint events. All patients were tracked till April 30, 2021. All occurrences were documented to determine the vessel-related clinical events. All adverse events were assessed by two experienced cardiologists, and if there was any discrepancies, a third physician was consulted to ensure the data was reliable. The primary outcome was major adverse cardiac and cerebrovascular events (MACCE), which was defined as a composite outcome of nonfatal myocardial infarction, revascularization (PCI and CABG), stroke, and cardiovascular death. The secondary outcomes were congestive heart failure, cardiogenic shock, malignant arrhythmia, and all-cause mortality. Unless an unmistakable non-cardiovascular cause was identified, all deaths were classified as cardiovascular deaths. Diagnosis of myocardial infarction was based on the fourth universal definition of myocardial infarction [29].

### 2.6 Statistical Analysis

SPSS22 software (IBM Corp., Armonk, NY, USA), STATA version 16.0 software (Stata Corp, College Station, Texas, USA), and R programming language version 3.3.1 (R Foundation for Statistical Computing, Vienna, Austria) were used to perform statistical analysis. The baseline characteristics were outlined with median and interquartile ranges for continuous variables. and counts and percentages for categorical variables. Multivariable adjusted odds ratios (ORs) were used to assess the relationship between independent risk factors and CAC. The analyses were adjusted for age, sex, smoking, total cholesterol, triglyceride, LDL, HDL, systolic blood pressure, diastolic blood pressure, diabetes mellitus, and family history of CAD. In addition, we formed four groups based on the number of CAD risk factors (These risk factors have been enlisted and defined in the ‘CAD risk factors’ subsection). We analyzed the correlation between the number of risk factors and CAC utilizing unadjusted ORs and ORs that had been adjusted for age- and gender.

The log-rank test was used to evaluate the significance of the difference in survival for each age group and CAC group. We calculated the MACCE rate per 1000 person-years (with 95% CI) based on CAC groups with stratification by the number of risk factors and age groups. The MACCE rate per 1000 person-years (with 95% CI) based on stenosis with a luminal cross-sectional area was also assessed and was stratified by different age groups. The incidence rate ratio was calculated using the Poisson regression 1000 person-years approach to derive the relative risks. The nomogram was created using the RMS (Root Mean Square) package of R software, version 3.3.1 (R Foundation for Statistical Computing, Vienna, Austria). For MACCE prediction, ROC (Receiver Operating Characteristic) curve analysis was used to determine the optimal cutoff value of cardiovascular risk prediction model. Sensitivity, specificity, positive likelihood ratio (positive LR), negative likelihood ratio (negative LR), positive predictive value (PPV), negative predictive value (NPV), and diagnostic accuracy were determined from the optimal threshold by the Youden index.

## 3. Results

### 3.1 Baseline Characteristics

**Fig. 1.**
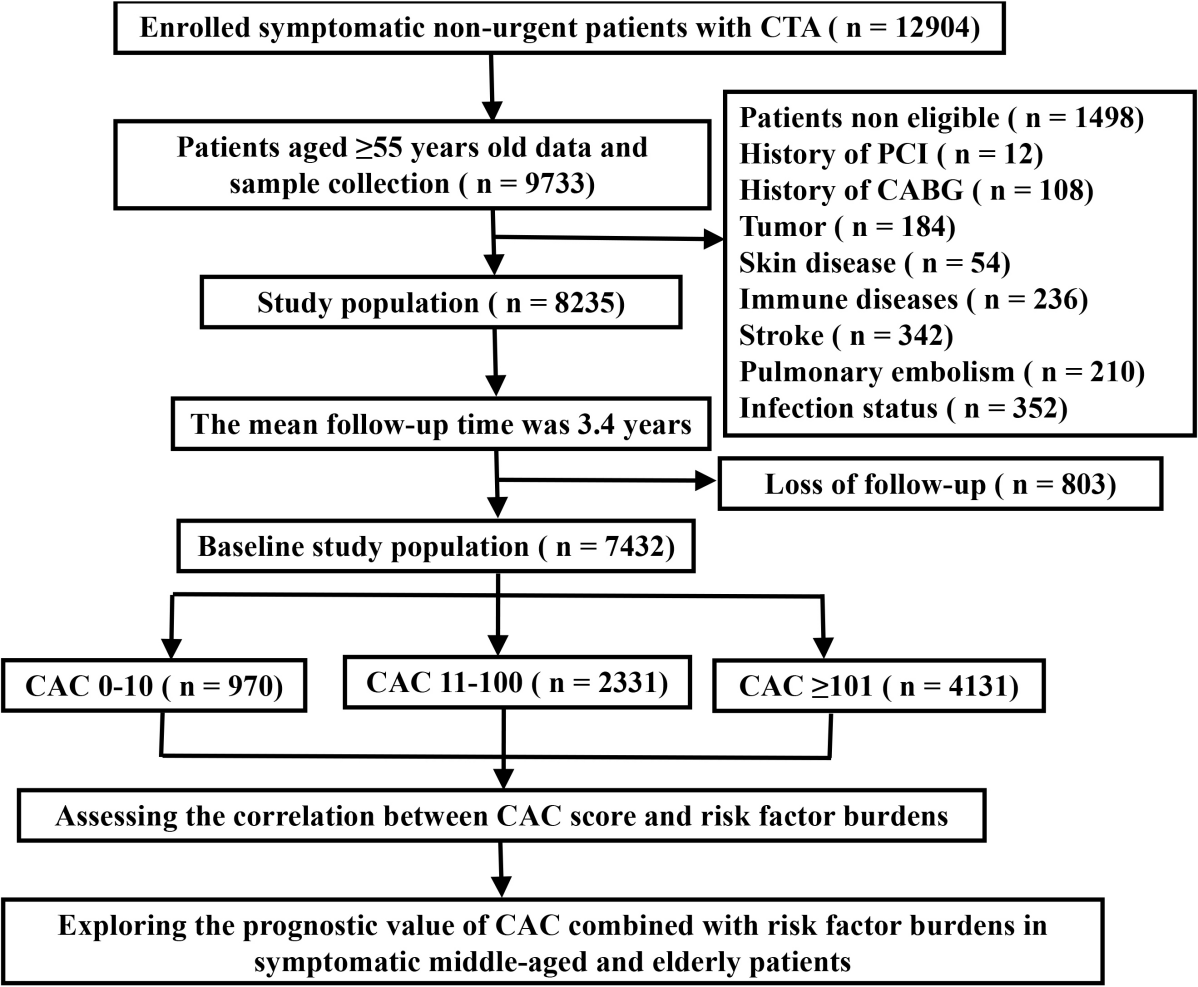
**Flow chart of the study procedure**. CTA, computed tomography angiography; PCI, percutaneous coronary intervention; CABG, coronary artery bypass graft; CAC, coronary artery calcium.

The study flow chart is presented in Fig. 1. A total of 7432 symptomatic patients were enrolled into the study. The baseline characteristics of those patients are shown in Table 1. The median age was 67 years (interquartile range [IQR]: 61–73 years). The number of patients with CAC 0–10 was 970 (13%, CAC 11–100 was 2331 (31%), and CAC ≥101 was 4131 (56%)). The median value and interquartile ranges of the CAC measurements for each group was 3 (IQR: 1.2–6), 42.4 (IQR: 23–66.7), and 379.6 (IQR: 197.4–827.1), respectively. 525 patients (7.1%) had 1 risk factor, 1275 patients (17.1%) had 2 risk factors, 1762 patients (23.7%) had 3 risk factors, and 3870 patients (52.1%) had 4 or more risk factors. In the total 7432 patients, 553 (7.4%) of whom had no CAD, 3889 (52.3%) nonobstructive CAD, and 2990 (40.3%) had obstructive CAD. The median duration of follow-up was 3.4 years (interquartile range: 1.8–5.4 years). A total of 478 patients (6.4%) had MACCE.

**Table 1.** **Baseline characteristics of middle-aged and elderly (≥55 Year of age)**.

		All	CAC 0–10	CAC 11–100	CAC ≥101	*p* value
Participants		7432 (100)	970 (13)	2331 (31)	4131 (56)	
Female		3773 (40)	480 (49)	974 (42)	1501 (36)	<0.0001
Age, years		67 (61–73)	64 (59–70)	65 (60–72)	69 (62–75)	<0.0001
	65 years > age ≥55 years	3028 (41)	515 (53)	1105 (47)	1408 (34)	0.049
	75 years > age ≥65 yaers	2838 (38)	330 (34)	854 (37)	1654 (40)	<0.0001
	age ≥75 years	1566 (21)	125 (13)	372 (16)	1069 (26)	0.004
Current smoking		2122 (29)	210 (22)	630 (27)	1282 (31)	<0.0001
Plasma parameters						
	TC	4 (3.2–4.7)	4.2 (3.4–4.9)	4.1 (3.4–4.8)	3.8 (3.1–4.6)	<0.0001
	TG	1.5 (1.1–2.2)	1.5 (1.0–2.2)	1.5 (1.1–2.3)	1.5 (1.1–2.2)	0.018
	LDL–C	2.6 (1.9–3.3)	2.8 (2.0–3.3)	2.7 (2.1–3.3)	2.5 (1.9–3.2)	<0.0001
	HDL–C	1.1 (0.9–1.3)	1.1 (1.0–1.4)	1.1 (0.9–1.4)	1.1 (0.9–1.3)	<0.0001
SBP, mmHg		130 (120–140)	126 (119–140)	130 (120–140)	130 (120–130)	0.039
DBP, mmHg		77 (70–81)	77 (70–82)	77 (70–83)	76 (70–80)	<0.0001
BMI, kg/$m^{2}$		25.7 (23.5–28.1)	25.6 (23.4–28.5)	25.6 (23.5–28.1)	25.8 (23.5–28.1)	0.851
Diabetes mellitus		2446 (33)	247 (25)	682 (29)	1517 (37)	<0.0001
Family history of CAD		872 (12)	116 (12)	280 (12)	476 (12)	0.545
CAC		134 (31.8–439.3)	3 (1.2–6)	42.4 (23–66.7)	379.6 (197.4–827.1)	<0.0001
Medication history						
	Antiplatelet aggregation	3044 (41)	366 (38)	887 (38)	1791 (43)	0.002
	CRM	3803 (51)	494 (51)	1149 (49)	2160 (52)	0.068
	CCB	1841 (25)	228 (24)	598 (26)	1015 (25)	0.387
	ACEI/ARB	1872 (25)	227 (23)	592 (25)	1053 (25)	0.388
	Nitroglycerin	484 (7)	46 (5)	136 (6)	299 (17)	0.008
	Beta–blocker	2244 (30)	288 (30)	674 (29)	1282 (31)	0.191

Values are n (%), %, or median (interquartile range), TC, total cholesterol; TG, triglyceride; LDL–C, low-density lipoprotein cholesterol; HDL–C, high-density lipoprotein cholesterol; SBP, systolic blood pressure; DBP, diastolic blood pressure; BMI, body mass index; CAC, coronary artery calcium; CAD, coronary artery disease; ACEI, angiotensin converting enzyme inhibitors; ARB, angiotensin receptor blockers; CRM, cholesterol-reducing medication; CCB, calcium-channel blocker.

### 3.2 Interplay between Age and CAC Score

**Fig. 2.**
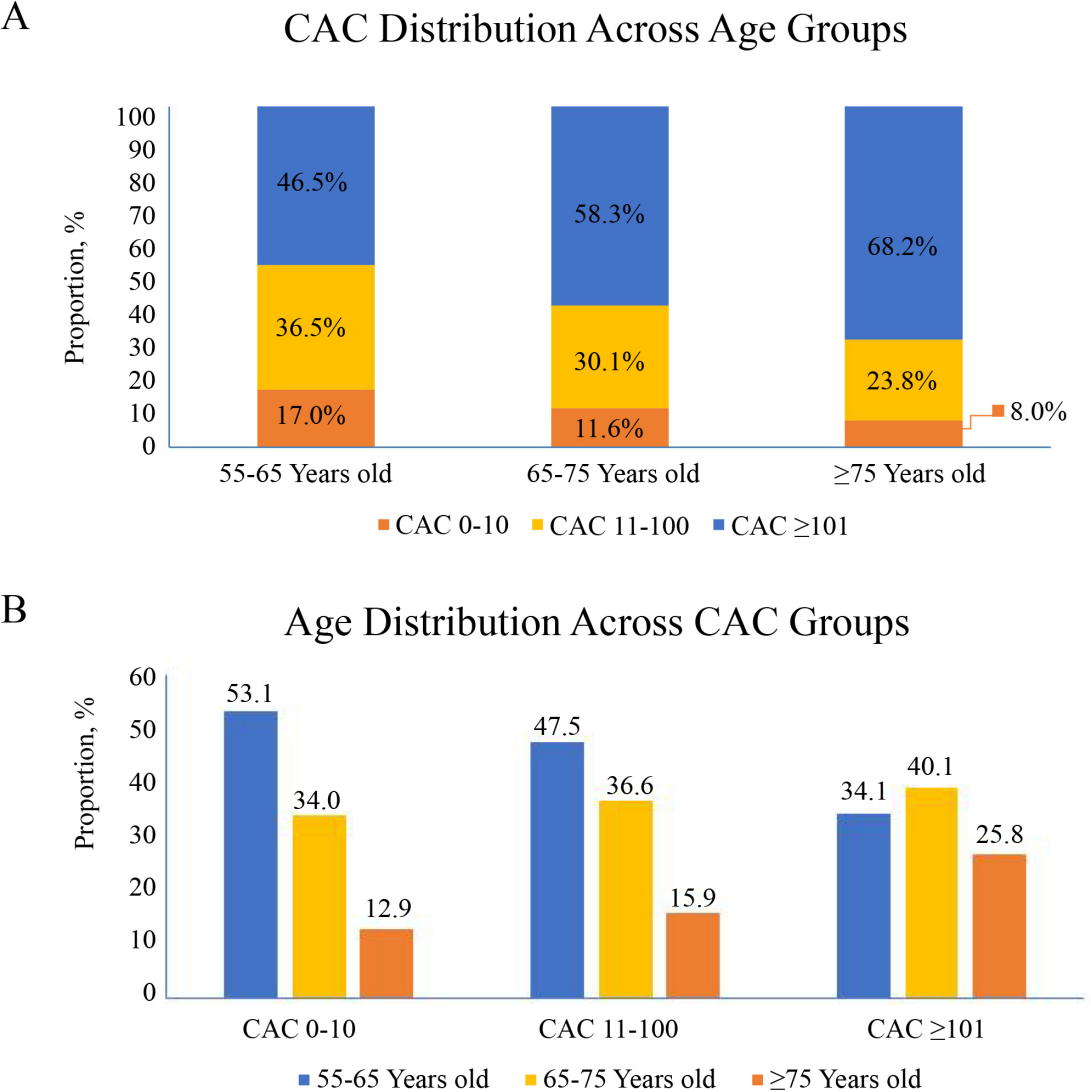
**Different age proportions in different CAC score groups in middle-aged and elderly patients (≥55 Years old)**. (A) CAC distribution across of age. (B) Age distribution across of CAC. CAC, coronary artery calcium.

The percentage of CAC ≥101 increased with increasing age (Fig. 2A). The percentage of CAC ≥101 increased from the 55–65 to ≥75-year-old group from 46.5% to 68.2% (relative increase, 21.7%). By comparison, the proportion of CAC 0–10 significantly declined from 17.0% to 8.0% between the 55–65-year-old group and the ≥75-year-old group. Additionally, as the CAC score increased, the proportion of patients in the ≥75-year-old group increased from 12.9% to 25.8%, compared to the 55–65-year-old group (Fig. 2B).

### 3.3 Relationship between the Number of Risk Factors and CAC in Symptomatic Patients Aged over 55 Years

**Fig. 3.**
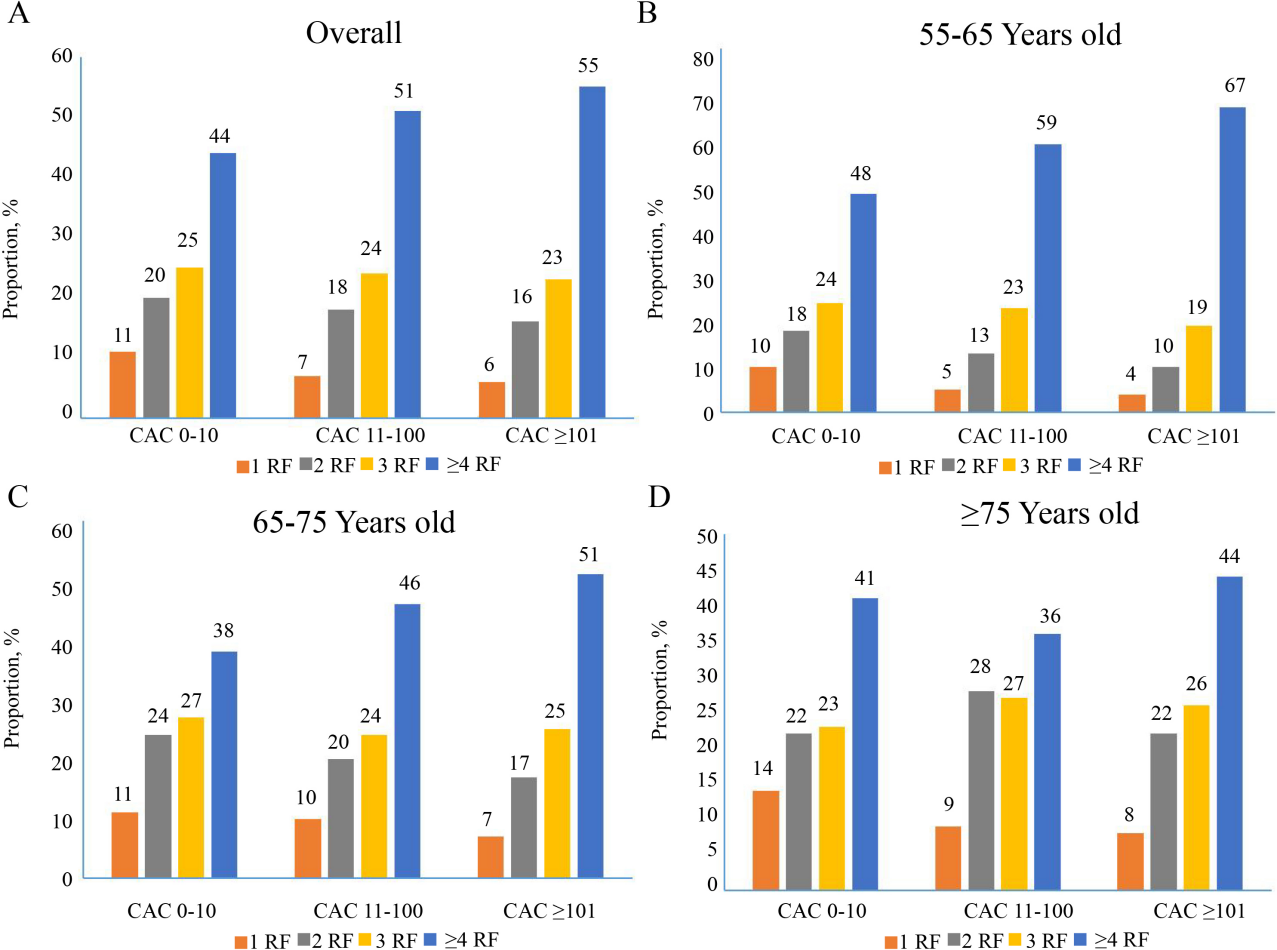
**Interplay between age groups and increasing CAC score for the presence of risk factor burden in middle-aged and elderly patients (≥55 Years old)**. (A) Risk factors distribution in total population. (B) Risk factors distribution in 55–65-year-old group. (C) Risk factors distribution CAC in 65–75-year-old group. (D) Risk factors distribution in ≥75-year-old group. CAC, coronary artery calcium; RF, risk factors.

Older age, LDL-C, and diabetes mellitus (DM) were the risk factors for the higher CAC scores, in comparison to the lower CAC scores (all ORs >1 and *p* values < 0.05, Table 2). As the number of CAD risk factors increased, the increasing trend of ORs was observed in each CAC score group (**Supplementary Table 1**). The proportion of risk factor ≥4 in symptomatic patients aged over 55 years increased in correlation with the CAC scores (Fig. 3A) and the similar tendencies were observed among the different age subgroups (Fig. 3B–D).

**Table 2.** **Odds ratio for presence of CAC ≤10 *Versus* CAC >10 and CAC 0–10, CAC 11–100 versus CAC ≥101 according to standard risk factors in middle-aged and elderly patients ≥55 years of age**.

CVD risk factors	CAC ≤10 versus CAC >10 Odds Ratio 95% CI	*p* value	CAC 0–10 versus CAC ≥101 Odds Ratio 95% CI	*p* value	CAC 11–100 versus CAC ≥101 Odds Ratio 95% CI	*p* value
Age	2.291 (1.621–3.238)	<0.001	2.896 (2.032–4.127)	<0.001	1.944 (1.564–2416)	<0.001
Female	0.685 (0.521–0.899)	0.006	0.607 (0.456–0.808)	0.001	0.759 (0.621–0.929)	0.008
Smoking	1.173 (0.876–1.570)	0.285	1.202 (0.889–1.626)	0.232	1.073 (0.877–1.313)	0.492
TC	0.561 (0.374–0.842)	0.005	0.408 (0.258–0.645)	<0.001	0.648 (0.463–0.907)	0.011
TG	1.004 (0.793–1.271)	0.976	1.016 (0.791–1.303)	0.903	0.969 (0.815–1.151)	0.715
LDL–C	1.697 (1.051–2.739)	0.031	2.299 (1.350–3.913)	0.002	1.446 (1.003–2.086)	0.048
HDL–C	0.807 (0.581–1.120)	0.200	0.783 (0.551–1.112)	0.172	0.893 (0.686–1.162)	0.400
SBP	0.997 (0.990–1.004)	0.403	0.996 (0.989–1.003)	0.282	0.997 (0.992–1.002)	0.198
DBP	1.075 (0.728–1.588)	0.717	0.957 (0.635–1.442)	0.833	0.721 (0.549–0.946)	0.018
BMI	1.002 (0.973–1.033)	0.879	1.000 (0.969–1.032)	0.990	0.993 (0.971–1.015)	0.517
DM	1.462 (1.143–1.870)	0.003	1.723 (1.333–2.226)	<0.001	1.501 (1.262–1.786)	<0.001
FH of CAD	0.943 (0.697–1.277)	0.705	0.868 (0.631–1.194)	0.384	0.939 (0.748–1.179)	0.586

DM, diabetes mellitus; FH, family history; other abbreviations as in Table 1.

### 3.4 Relationship between Normal, Nonobstructive, Obstructive Disease and Age in Symptomatic Patients Aged over 55 Years

**Fig. 4.**
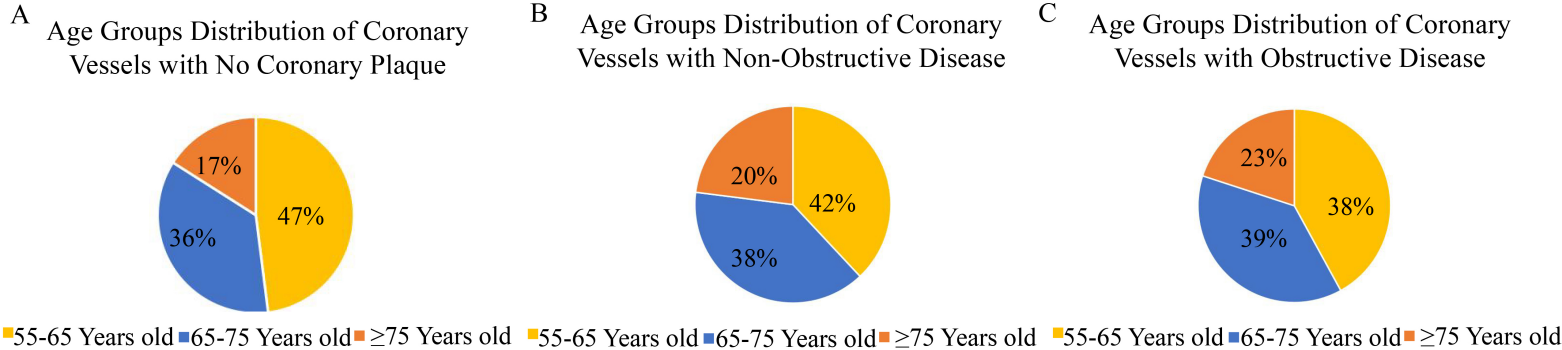
**Age groups distribution across different type of coronary artery disease**. (A) Age distribution of patients without coronary artery disease. (B) Age distribution of patients with non-obstructive coronary artery disease. (C) Age distribution of patients with obstructive coronary artery disease.

**Fig. 5.**
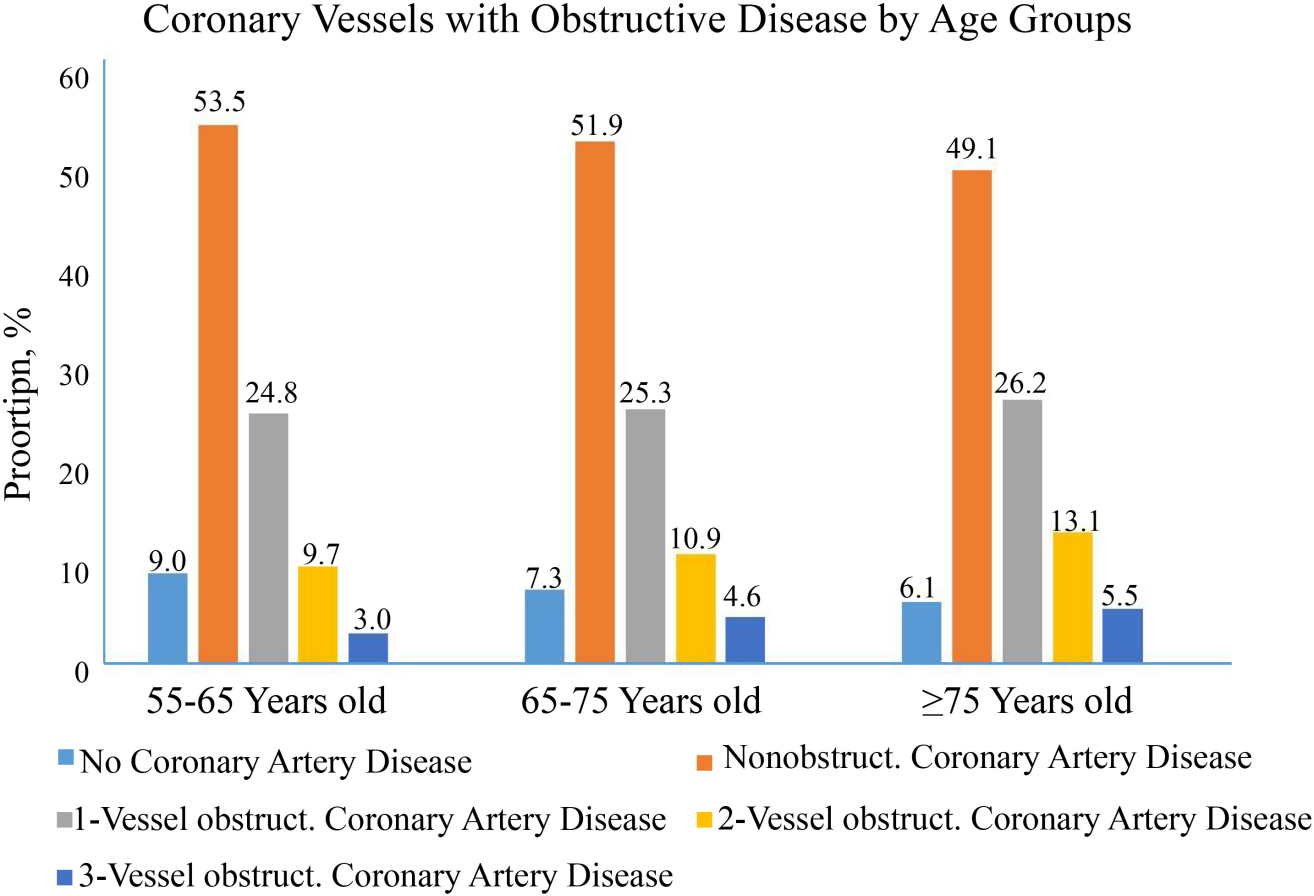
**Age distribution of coronary vessels with obstructive coronary artery disease**.

Plaque characteristics in relation to age appeared to vary considerably. For example, middle-aged and elderly women had 1.816 times (95% CI: 1.150–2.867) higher chances of having calcified plaques, while the inverse was true for diabetes mellitus (**Supplementary Table 2**). Overall, Patients aged ≥75 years accounted for 17% in the patients without CAD, 20% in the patients with nonobstructive CAD, and 23% in the patients with obstructive CAD (Fig. 4). The detection of non-obstructive CAD was similar among the three age groups (53.5% *vs* 51.9% *vs* 49.1%), while the percentage of obstruction CAD increased as the age increased (Fig. 5).

### 3.5 Interplay of Risk Factors and CAC, Age and CAC, Age and Different Types of CAD for Risk for MACCE in Patients Aged over 55 Years Old

**Fig. 6.**
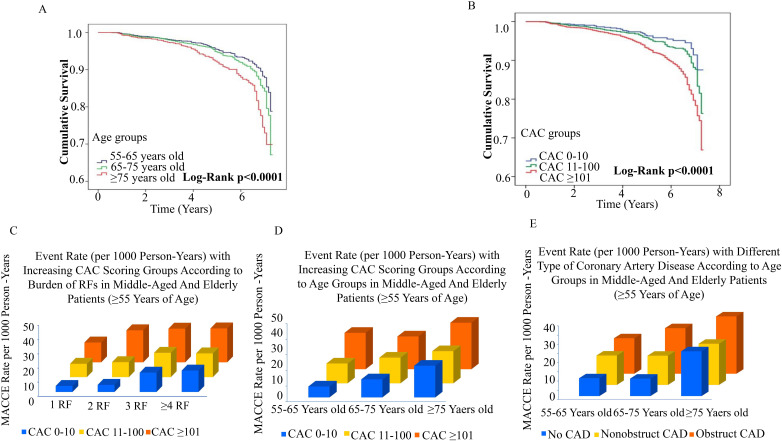
**Interplay of risk factors and CAC, age and CAC, age and different types of coronary artery disease for risk of MACCE in middle-aged and elderly patients (≥55 Years old)**. (A) Cumulative survival rates for different age groups. (B) Cumulative survival rates for different CAC groups. (C) Events rate (per 1000 person years) with increasing CAC score according to the burden of risk factors. (D) Events rate (per 1000 person years) with increasing CAC score according to different age groups. (E) Events rate (per 1000 person years) with different type of coronary artery disease according to different age groups. CAC, coronary artery calcium; MACCE, major adverse cardiac and cerebrovascular events; CAD, coronary artery disease.

**Fig. 7.**
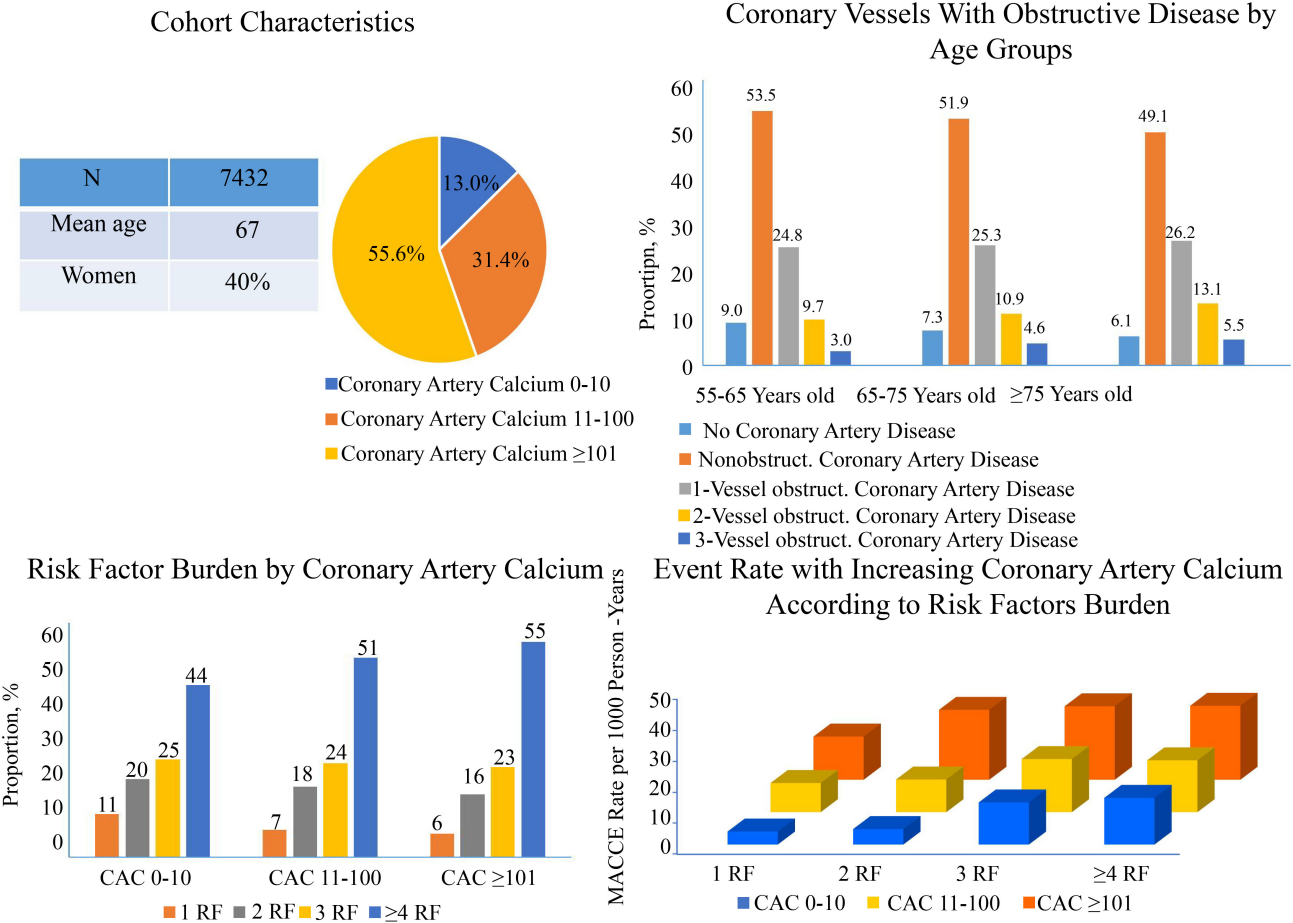
**Interplay between age group and coronary artery calcium in middle-aged and elderly symptomatic patients**. CAC, coronary artery calcium; RF, risk factors.

**Fig. 8.**
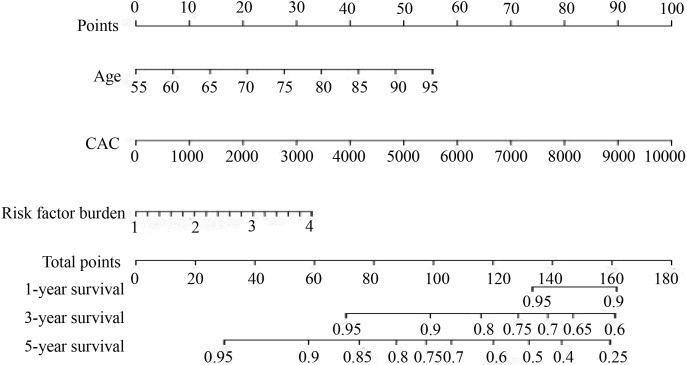
**The nomogram was used to calculate predicted MACCE risk for symptomatic patients ≥55 years old**. The term “Points” represents the score of each variable under different values, “Total Points” means the total score of the collection after the sum of the corresponding individual fractions of all variables. To use the nomogram, first draw a vertical line to the top points row to assign points for each variable; then, add the points from each variable together and drop a vertical line from the total points row to obtain the 1-year survival, 3-year survival, 5-year survival, and median survival time (in years). CAC, coronary artery calcium.

**Fig. 9.**
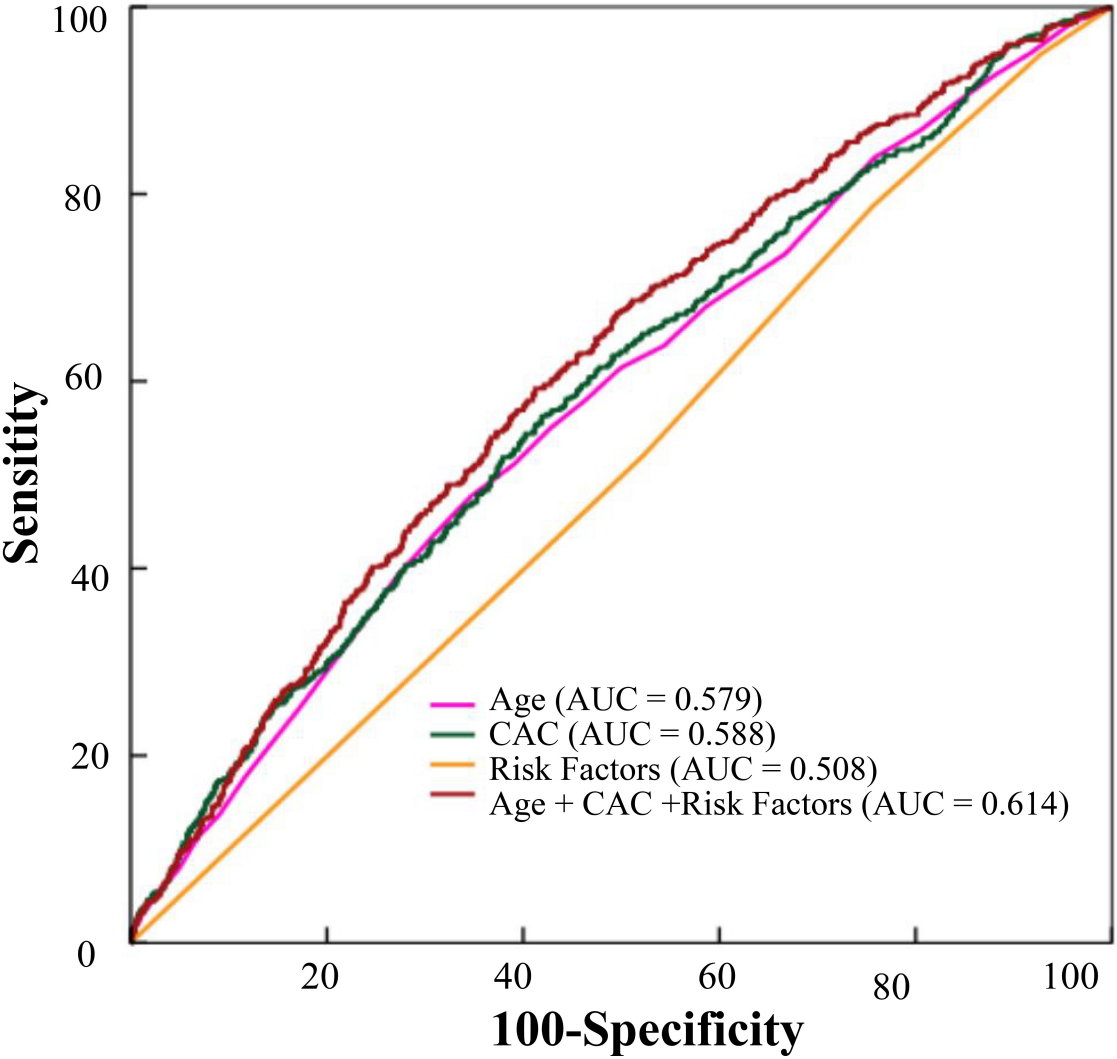
**The predictive effects of age, CAC, risk factors, CAC combined with age and risk factors on MACCE were analyzed**. CAC, coronary artery calcium; MACCE, major adverse cardiac and cerebrovascular events.

The cumulative incidence of survival decreases with increasing age and CAC scores (*p *< 0.0001) (Fig. 6A,B). The risk of MACCE increased with increase of CAC score and the number of risk factors (Fig. 6C, **Supplementary Table 3**, Fig. 7). The risk of MACCE was significantly higher in patients with ≥4 risk factors and CAC ≥101 than in those with 1 risk factors and CAC 0–10. There were 1.71 times higher event rates in patients with CAC ≥101 and ≥4 risk factors than in patients with CAC ≥101 and 1 risk factor (*p* = 0.044) (**Supplementary Table 4**). As the number of CAD risk factors increased, the MACCE survival rate decreased significantly (**Supplementary Fig. 1**). As CAC scores increased, the risk of MACCE events rose, and the MACCE rate was highest in those aged ≥75 years and those CAC ≥101 (Fig. 6D and **Supplementary Table 5**). Similar trends were observed for the different types of CAD. For patients with non-obstructive CAD, the incidence of MACCE events gradually increases with age. As an illustration, in patients with non-obstructive CAD, the risk of MACCE in patients aged ≥75 years was 2.62 times (95% CI: 1.83–3.74; *p *< 0.0001) higher than that in patients aged 55–65 years (Fig. 6E and **Supplementary Table 6**). A predictive MACCE-related prognostic nomogram was established using the results of the multivariate analysis to predict the 1-, 3-, and 5-year overall survival. As shown, this nomogram was able to assess several variables to predict a patient outcome including age, CAC and risk factor burdens (Fig. 8). Further using the Youden index test, we found that CAC combined with age and risk factors had the highest predictive power for MACCE (AUC = 0.614) (Fig. 9). The details of statistical results about each MACCE risk prediction model were showed in **Supplementary Table 7**.

## 4. Discussion

The present study evaluated the long-term clinical outcomes and predictors of mortality among a large cohort of patients over 55 years of age who had a possible diagnosis of CAD and underwent first-line CTA. We conducted a depth analysis of the correlation cardiovascular risk factors, age, and CAC scores and combined them for future prediction of MACCE. Four main conclusions were drawn from this study. Firstly, the increase of risk factor burdens for middle-aged and elderly individuals with CAC ≥101 was linked to a heightened risk of MACCE, indicating that traditional risk factors are a contributing factor in atherosclerotic disease among this population. Consequently, utilizing risk factors can help to determine which patients would benefit from CTA testing at a younger age. Secondly, even with a low CAC score, patients aged ≥75 years were at a significantly higher risk of MACCE compared to younger patients. Assessing risk factors in elderly individuals is particularly important, especially in those with low CAC or CAC = 0. Thirdly, in non-obstructive CAD, the risk of MACCE increases with age. Therefore, more attention should be paid to middle-aged and elderly patients with non-obstructive CAD. Fourth, CAC combined with risk factor burdens and age can improve the predictive value of MACCE (AUC = 0.614). It is preferable to consider the patient’s age, risk factors, and CAC scores when attempting to predict cardiovascular events in those middle-aged and elderly CAD-suspected patients. Early preventive interventions for these individuals could reduce the risk of cardiovascular events.

### 4.1 Risk Factors and CAC in the Middle-Aged and Elderly

Currently, the existing evidence on the relationship between risk factors and CAC in middle-aged and elderly patients is limited. A prospective study on a large multi-ethnic cohort of individuals aged 45–84 years who had no pre-existing clinical cardiovascular disease revealed that those with CAC = 0 had a low 10-year risk of event rate, but individuals with CAC ≥100 had a higher risk of event rate which was consistently above 7.5% [18]. A cohort study of 12,441 Korean patients with an average age of 52 years demonstrated that individuals with diabetes mellitus experienced greater CAC progression than those without diabetes mellitus, and diabetes had an incremental effect on CAC progression [30]. Previous research has suggested that an increase in traditional CAD risk factors is associated with an increase in CAC [7]. Across the spectrum of risk factor burden, a greater CAC score is strongly associated with an increased risk of long-term all-cause mortality, as well as a larger proportion of death caused by CVD and CAD [16, 31, 32, 33]. Our research adds to the preceding study, verifying the association between traditional risk factors and CAC scores, demonstrating the potential clinical application of risk factors. In addition, our research revealed that the middle-aged and elderly patients with four or more risk factors had 1.87 times higher odds of having CAC ≥101 compared to those with one risk factor. Notably, it is worth mentioning that 1 in 2 middle-aged and elderly adults with CAC ≥101 had ≥4 risk factors. Combing the results, it became evident that the risk factors are critical in predicting the probability of developing coronary calcification in middle-aged and elderly adults.

### 4.2 CAC and Cardiovascular Risk in the Middle-Aged and Elderly

Stenosis of a vessel blocking the bloodstream leads to CAD, resulting in underperfusion of the heart region due to blocked vessel. Distinct types of CAD are caused by diverse degrees of coronary obstruction. However, obstructive CAD is associated with higher myocardial infarction rates [34, 35, 36]. Our research showed that when the number of CAD risk factors increased, the rate of obstructive CAD also increased. Specifically, we found that the rate of obstructive CAD was 5.7% among those with CAC 0–10 and 1 risk factor, while it was 16.5% among those with CAC 0–10 and 4 or more risk factors. Clinicians should alert those with a lower CAC score but higher risk factors to their possible health hazards. The MESA study indicated that cardiovascular events significantly increased with increasing CAC in middle-aged and elderly individuals [16]. Our study showed that the incidence of MACCE was lower in the group with CAC 0–10, but the incidence of MACCE was higher in the group with CAC 0–10 and ≥4 risk factors than in the group with CAC 0–10 and 1 risk factor, which indicated that risk factor burden can increase the risk of MACCE. Previous studies have shown that increased CAC was associated with a higher risk of future cardiovascular events in asymptomatic patients [37, 38]. In our study, the number of patients in CAC 0–10, CAC 11–100, and CAC ≥101 group was 970 (13%), 2331 (31.4%), and 4131 (55.6%), respectively. At a mean follow-up of 3.4 years period, the incidence of MACCE was 3.9%, 5.2%, and 7.7%, respectively. We found that combining CAC with age and risk factors improves the predictive value of MACCE events. The AUC ranged from 0.59 to 0.64 indicating that this model’s predictive ability for MACCE was moderate. These questions raised by this study warrant further investigation of a better risk score model.

### 4.3 Study Limitations

Although we adjusted for gender in our analyses, potential unadjusted residual confounding factors may still exist. All the patients in our study were symptomatic, which may reduce the generalizability of the results to asymptomatic patients. When assessing symptomatic patients with CTA, it cannot exclude a degree of bias, as those deemed to be high-risk (e.g., aged above 75 years) may be more likely to be referred for invasive angiography. Therefore, this may reduce the generalizability of the results to the elderly and higher-risk groups. Age, risk factors, and CAC score are closely related and may affect each other with an increased severity of coronary artery disease. However, our study was based on real world data which is a major strength of this study. In addition, we obtained information on the CAC score and CT angiography results.

## 5. Conclusions

The study revealed that for symptomatic patients aged ≥55 years, the greater the age, CAC scores, and risk factor burden, the more likely it was to lead to a future MACCE. By combining CAC scores, age, and risk factors, it is possible to more accurately predict the outcomes of symptomatic middle-aged and elderly patients. These results highlight the need to consider risk factors, CAC scores, and age when evaluating the risk of MACCE in middle-aged and elderly adults.

## Data Availability

All the data were presented in the main paper. The data that support the findings of this study are available on request from the corresponding author. All authors take responsibility for the integrity of the data and the accuracy of the data analysis.

## Supplementary Material

Supplementary material associated with this article can be found, in the online version, at https://doi.org/10.31083/j.rcm2406158.
