# Supplementary material for: Interplay between Risk Factors and Coronary Artery Calcium in Middle-Aged and Elderly Symptomatic Patients
Source: Rev Cardiovasc Med. 2023 Jun 6;24(6):158. doi: 10.31083/j.rcm2406158 (PMC11264111; doi:10.31083/j.rcm2406158)
Supplement: Supplementary file 1 [file 2153-8174-24-6-158-s1.docx]

**Supplement Table 1. Odds ratio for presence of CAC 11-100 and CAC≥101 versus CAC 0-10 according to number of risk factors in middle-aged and elderly patients ≥55 years of age**

| Risk factor burden | CAC 11-100  Odds Ratio 95% CI  (crude) | CAC 11-100  Odds Ratio 95% CI (age- and sex-adjusted) | CAC≥101  Odds Ratio 95% CI  (crude) | CAC ≥101  Odds Ratio 95% CI (age- and sex-adjusted) |
| --- | --- | --- | --- | --- |
| 1 (525) | ref | ref | ref | ref |
| 2 (1275) | 1.332 (0.990-1.793) | 1.242 (0.916-1.682) | 1.378 (1.044-1.818) | 1.147 (0.858-1.534) |
| 3 (1762) | 1.465 (1.100-1.953) | 1.360 (1.007-1.835) | 1.626 (1.245-2.153) | 1.337 (1.002-1.783) |
| ≥4 (3870) | 1.768 (1.352-2.312) | 1.615 (1.196-2.180) | 2.204 (1.717-2.830) | 1.873 (1.403-2.499) |

CAC = coronary artery calcium; CAD = coronary artery disease; CI = confidence interval

**Supplement Table 2. Odds ratio for presence of mixed plaque versus non-calcified plaque and calcified plaque versus mixed plaque according to coronary artery disease risk factors in middle-aged and elderly patients ≥55 years of age.**

| CAD risk factors | mixed plaque versus  non-calcified plaque  Odds Ratio 95％CI | calcified plaque versus  mixed plaque  Odds Ratio 95％CI |
| --- | --- | --- |
| Age | 1.041 (0.986-1.100) | 0.952 (0.928-0.978) |
| Female | 0.410 (0.157-1.073) | 1.816 (1.150-2.867) |
| Smoking | 1.309 (0.430-3.980) | 0.924 (0.564-1.513) |
| Total cholesterol | 0.494 (0.136-1.785) | 0.813 (0.376-1.754) |
| Triglyceride | 1.356 (0.591-3.109) | 0.979 (0.664-1.443) |
| LDL cholesterol | 0.910 (0.225-3.678) | 0.790 (0.328-1.898) |
| HDL cholesterol | 1.139 (0.370-3.505) | 1.243 (0.743-2.080) |
| Systolic blood pressure | 1.036 (1.008-1.065) | 0.987 (0.975-0.999) |
| Diastolic blood pressure | 0.499 (0.128-1.946) | 1.291 (0.668-2.496) |
| Body mass index | 1.044 (0.937-1.165) | 0.937 (0.889-0.988) |
| Diabetes mellitus | 1.020 (0.445-2.334) | 0.632 (0.414-0.964) |
| Family history of CAD | 0.947 (0.347-2.584) | 0.821 (0.487-1.384) |

CAD = coronary artery disease; CI = confidence interval, HDL = High-density lipoprotein; LDL = Low-density lipoprotein


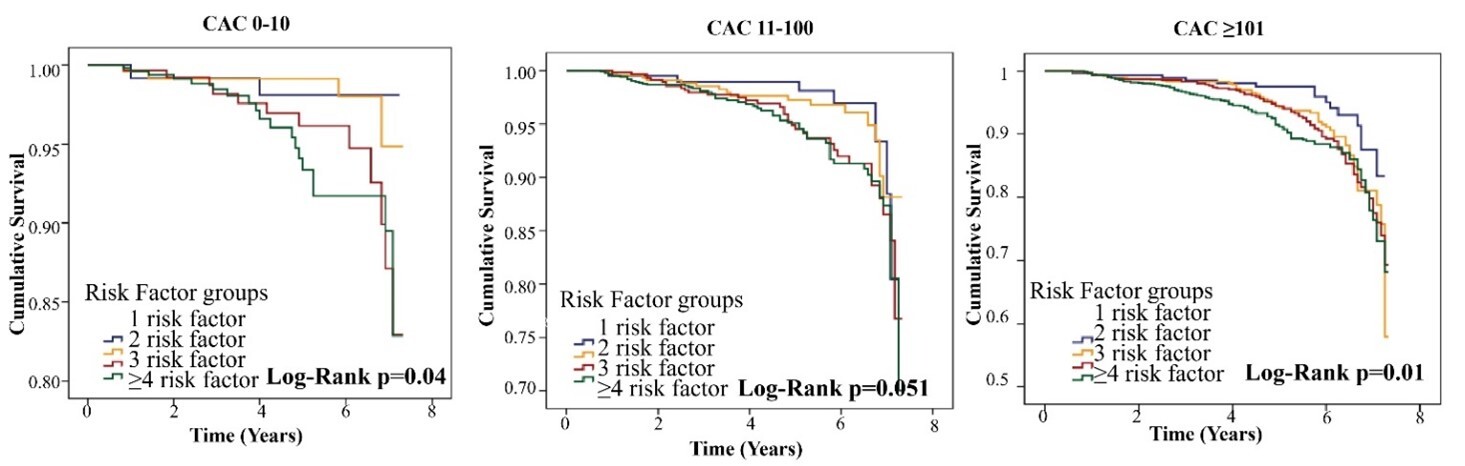


**Supplement Figure 1. Cumulative survival rates for different burden of risk factors in each CAC group.**

**Supplement Table 3. Relationship Between Coronary Artery Calcium and Coronary artery disease in Middle-Aged and Elderly (≥55 Years Old)**

|  | | MACCEs | | | |
| --- | --- | --- | --- | --- | --- |
| CAC burden |  | | Event Rate per 1000 Person-Years | Unadjusted  Hazard Ratio | Adjusted  Hazard Ratio* |
| CAC 0-10 | 970 (38) | | 10.98 (4.44-17.56) | Ref. | Ref. |
| CAC 11-100 | 2331 (122) | | 14.99 (10.06-19.92) | 1.355 (0.934-1.965) | 1.271 (0.875-1.847) |
| CAC ≥101 | 4131 (318) | | 22.93 (18.50-27.50) | 2.045 (1.450-2.885) | 1.724 (1.217-2.442) |

Values are n (%), unless otherwise indicated. *Adjusted for age and sex. MACCE = major adverse cardiovascular and cerebrovascular event; CAC = coronary artery calcium

**Supplement Table 4. Incidence rate ratio with increasing coronary artery calcium scoring groups according to burden of risk factors in middle-aged and elderly patients ≥55 years of age**

| Risk factor burden | MACCEs dependent on CAC burden | | | | | |
| --- | --- | --- | --- | --- | --- | --- |
|  | CAC 0-10  Event rate per  1000 person-years | p value | CAC 11-100  Event rate per  1000 person-years | p value | CAC≥101  Event rate per  1000 person-years | p value |
| 1 | ref |  | ref |  | ref |  |
| 2 | 1.20 (0.22-6.58) | 0.835 | 1.12 (0.47-2.71) | 0.796 | 1.63 (0.92-2.88) | 0.091 |
| 3 | 3.23 (0.72-14.46) | 0.124 | 1.83 (0.81-4.11) | 0.149 | 1.70 (0.98-2.96) | 0.058 |
| ≥4 | 3.59 (0.84-15.31) | 0.085 | 1.78 (0.81-3.89) | 0.148 | 1.71 (1.01-2.92) | 0.044 |

CAC = coronary artery calcium; MACCE = major adverse cardiovascular and cerebrovascular event

**Supplement Table 5. Incidence rate ratio with increasing coronary artery calcium scoring groups according to age groups in middle-aged and elderly patients ≥55 years of age.**

| Age groups | MACCEs dependent on CAC burden | | | | | |
| --- | --- | --- | --- | --- | --- | --- |
|  | CAC 0-10  Event rate per  1000 person-years | p value | CAC 11-100  Event rate per  1000 person-years | p value | CAC≥101  Event rate per  1000 person-years | p value |
| 55-65 (3927) | ref |  | ref |  | ref |  |
| 65-75 (3616) | 1.87 (0.89-3.93) | 0.099 | 1.42 (0.95-2.13) | 0.088 | 1.02 (0.78-1.33) | 0.899 |
| ≥75 (2070) | 12.65 (6.74-23.75) | <0.0001 | 1.81 (1.13-2.91) | 0.014 | 1.46 (1.11-1.93) | 0.007 |

CAC = coronary artery calcium; MACCE = major adverse cardiovascular and cerebrovascular event

**Supplement Table 6. Incidence rate ratio with the type of coronary artery disease according to age groups in middle-aged and elderly patients ≥55 years of age**

| Age groups | MACCEs dependent on the type of coronary artery disease | | | | | |
| --- | --- | --- | --- | --- | --- | --- |
|  | No CAD  Event rate per  1000 person-years | p value | Non-obstruct CAD Event rate per 1000 person-years | p value | Obstruct CAD  Event rate per 1000 person-years | p value |
| 55-65 (3927) | ref |  | ref |  | ref |  |
| 65-75 (3616) | 1.61 (0.70-3.72) | 0.267 | 1.69 (1.20-2.37) | 0.003 | 0.92 (0.68-1.24) | 0.579 |
| ≥75 (2070) | 1.95 (0.74-5.12) | 0.176 | 2.62 (1.83-3.74) | <0.0001 | 1.28 (0.93-1.76) | 0.128 |

CAD = coronary artery disease; MACCE = major adverse cardiovascular and cerebrovascular event

**Supplement Table 7. Evaluating the predictive value of CAD risk prediction model for MACCE.**

| Variables | ROC curve parameters | | | | | | | | |
| --- | --- | --- | --- | --- | --- | --- | --- | --- | --- |
|  | Cut-off | AUC | 95% CI | Sensitivity | Specificity | PPV (%) | NPV (%) | LR+ | LR- |
| Age | 71 | 0.579 | 0.552-0.605 | 48 | 65 | 42 | 40 | 1.37 | 1.25 |
| CAC | 186 | 0.588 | 0.562-0.612 | 42 | 58 | 42 | 42 | 1.00 | 1.00 |
| Risk Factor | 3 | 0.508 | 0.482-0.534 | 79 | 24 | 77 | 78 | 1.04 | 1.14 |
| Age and CAC and Risk Factor | 18 | 0.614 | 0.588-0.639 | 59 | 59 | 50 | 50 | 1.44 | 1.44 |

ROC = receiver operating characteristic curve; AUC = area under the receiver operating characteristic.

PV = positive predictive value; NPV = negative predictive value; LR+ = positive likelihood ratio. LR- = negative likelihood ratio; MACCE = major adverse cardiovascular and cerebrovascular event.
